# Acute Blindness as a Complication of Severe Acute Respiratory Syndrome Coronavirus-2

**DOI:** 10.7759/cureus.16857

**Published:** 2021-08-03

**Authors:** Fatima Zahra Mabrouki, Rachid Sekhsoukh, Faiza Aziouaz, Yassine Mebrouk

**Affiliations:** 1 Ophthalmology, Faculty of Medicine and Pharmacy, Mohammed VI University Hospital, Oujda, MAR; 2 Neurology, Faculty of Medicine and Pharmacy, Mohammed VI University Hospital, Oujda, MAR

**Keywords:** sars-cov-2, neurological manifestations, bilateral optic neuritis, acute blindness, post-covid-19 symptoms

## Abstract

Several neurological manifestations can occur in the acute phase or in post-infection severe acute respiratory syndrome coronavirus 2 (SARS-CoV-2). In certain cases, they can even reveal the disease. Although some may be consequences of direct cellular viral invasion, many represent post-infectious inflammation mediated by autoimmune mechanisms. We report the case of a 60-year-old woman who was initially consulted for acute blindness without optic neuritis. Brain MRI revealed nonspecific demyelinating lesions without any radiological signs of optic neuritis. The patient underwent an exhaustive assessment and then the diagnosis of optic neuritis with a normal orbital MRI following a SARS-CoV-2 infection was reached.

## Introduction

The severe acute respiratory syndrome coronavirus-2 (SARS-CoV-2) is a new pandemic that emerged in China in December 2019 [1]. It may be related to several neurological manifestations that occur in the acute phase of SARS-CoV-2 or post-infection. In some cases, they can even reveal the disease [2]. Neurological involvement during a COVID infection may be related to either direct cellular viral invasion or post-infectious inflammation mediated by autoimmune mechanisms [3]. We report the case of a 60-year-old woman who was seen in consultation initially for ocular symptoms and then was discovered incidentally to have a SARS-CoV-2 infection on admission.

## Case presentation

A 60‐year‐old female presented to the emergency department in our hospital with acute bilateral eye vision loss. Her past medical history included an undocumented splenectomy, diabetes mellitus, and hypothyroidism. Two weeks prior to the onset of visual symptoms, she reported flu symptoms, including fever, myalgia, and dry cough. The patient neglected these symptoms and did not benefit from any treatment. Fifteen days later, the patient presented with a sudden drop in visual acuity in the left eye without pain, eye redness, or headache which became blinding after 24 hours with involvement of the contralateral eye after four days, associated with right hemibody weakness. Her admission parameters were as follows: pulse 94 beats per minute (bpm), blood pressure 135/88 mmHg, temperature 38.2°C, and oxygen saturations were 85% on room air.

Her neurological examination on admission revealed complete bilateral blindness with no perception of light. Pupil responses to light were abolished with horizontal nystagmus and unremarkable fundoscopic findings. No other obvious abnormal findings in cognitive function, cranial nerves, or neck rigidity were demonstrated. Motor system examination revealed normal bulk in all four limbs. There was hypotonia, most marked on the right side. Power was grade 4/5 in the right hemibody and 5/5 in the left side. There was no involuntary movement or seizures. Plantar reflexes showed bilaterally Babinski signs. All modalities of sensation were conserved. Clinical examination of other systems revealed no abnormalities She presented with breathlessness a few hours after her admission to the neurology department. She was then transferred to the intensive care unit following clinical deterioration and continued increasing oxygen requirements where she was started on non-invasive ventilation.

**Figure 1:**
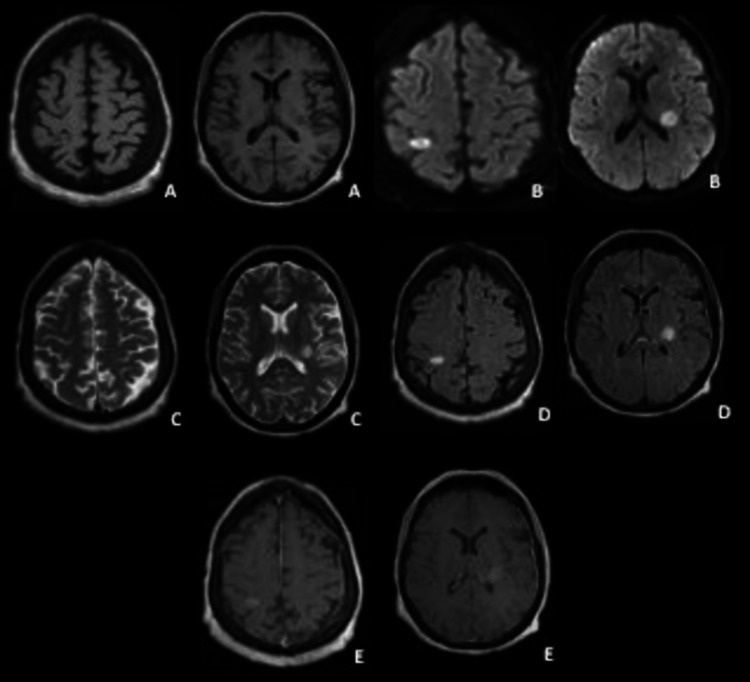
Brain magnetic resonance imaging (1.5 Tesla) A) T1 axial view; B) Diffusion axial view; C) T2 axial view; D) T2 fluid-attenuated inversion recovery (FLAIR) axial view; E) T1 axial view with gadolinium revealed right parietal nodular signal abnormality in the posterior limb of the internal capsule with enhancement after gadolinium

**Figure 2:**
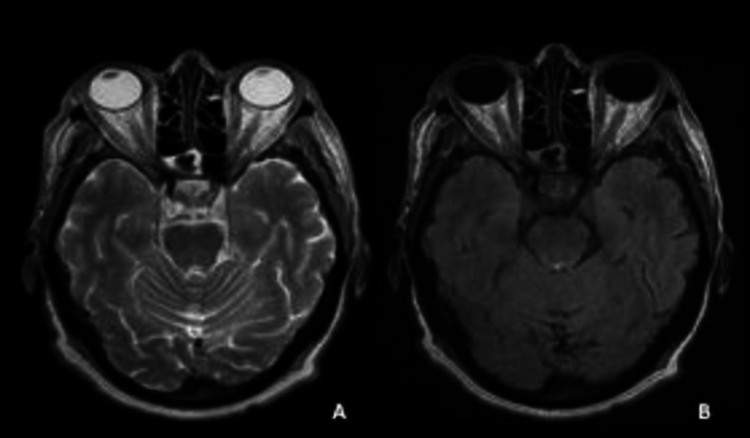
Orbital magnetic resonance imaging (1.5 Tesla) reveals absence of signal abnormalities on the optic nerve A) Axial T2 view; B) Axial T2 fluid-attenuated inversion recovery (FLAIR) view

The patient had magnetic resonance imaging (MRI) (1.5 Tesla) of the brain and orbits with and without contrast, which revealed right parietal nodular signal abnormality in the posterior limb of the internal capsule (Figures 1-2)". No intramedullary abnormal signal or enhancement was present.

**Figure 3:**
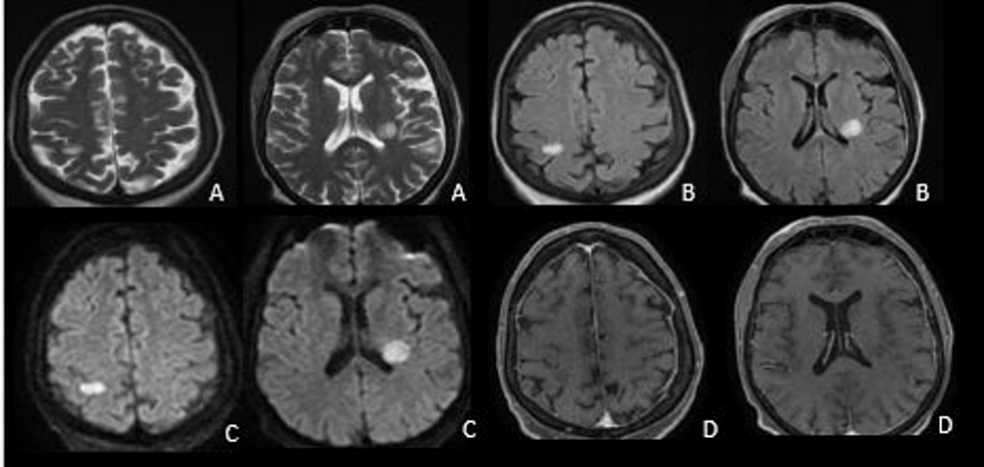
Brain magnetic resonance imaging (MRI) four weeks after admission showing stability of the lesion load with optic atrophy and disappearance of contrast enhancement A) T2 axial view; B) T2 fluid-attenuated inversion recovery (FLAIR) axial view; C) Diffusion axial view; D) T1 Axial view with gadolinium

Biochemical and cytological studies of the cerebrospinal fluid (CSF) showed an elevated protein count (1.41 g/L) and normal glucose without pleocytosis. No oligoclonal band was found in the CSF analysis. Blood and CSF cultures were negative. SARS-CoV-2 ribonucleic acid (RNA) in the CSF was not detected. She tested negative for myelin oligodendrocyte glycoprotein immunoglobulin G (MOG-IgG) and serum aquaporin-4 IgG antibodies. The serum paraneoplastic panel was assessed at the Mayo Clinic, and the serum angiotensin-converting enzyme (ACE) levels were normal. Blood tests showed a highly elevated C-reactive protein (CRP) at 300 mg/L with marked hyperleukocytosis (15,510/μl). Other laboratory results, such as copper, B12, and zinc levels, were checked and were within the reference range. Her nasopharyngeal swab reverse transcription-polymerase chain reaction (RT-PCR) was negative for SARS-CoV-2. COVID serology was positive for immunoglobulin M (IgM) and IgG antibodies. Other serological tests for syphilis, human immunodeficiency virus (HIV), human T-cell leukemia virus, types 1 and 2 (HTLV I/II), Epstein-Barr virus, cytomegalovirus, and hepatitis B and C were all negative. Serum ferritin (42.92 μ/L) and interleukin-6 levels (7.8 pg/mL) were normal. They were only measured on Day 5 of her admission (15 days after symptom onset). The patient reported no family history of cancer. Computed tomography (CT) imaging of the thorax, abdomen, and pelvis was done to assess possible malignancy, which was negative. The patient received seven sessions of plasmapheresis and motor physiotherapy sessions, with the prevention of thromboembolic complications by anticoagulant therapy. Her clinical evolution was stationary. A second control by encephalic and medullar MRI was performed and showed stability of the lesion load with disappearance of contrast enhancement with optic atrophy (Figure 3).

During her stay in intensive care, she benefited from an immunoglobulin cure (0.40 g/kg per day for five days) associated with corticosteroids. The evolution was marked by the remission of the respiratory discomfort, an improvement in the motor deficits on the right side (5/5 proximal, 4/5 distal), and the persistence of blindness. The patient was then discharged with regular neurology follow-up.

## Discussion

The occurrence of focal neurological deficits in SARS-CoV-2 infection is becoming increasingly recognized [4]. Several mechanisms can explain the occurrence of neurological manifestations during infection with SARS COV2: directly by neurotoxic action when the virus binds to the angiotensin-converting enzyme 2 (ACE2) receptor after reaching the nervous system by different routes or indirectly via a cytokine storm with immunological mediation, blood-brain barrier disruption, and by increased blood coagulation [5]. Immune complex deposition and molecular mimicry have been elucidated as one of the immunopathogenic processes involved in *Mycoplasma pneumoniae* post-infectious optic neuritis [6]. Activation of lymphocytes is another interesting mechanism by microbial superantigens that can initiate immunopathogenesis and lead to autoimmune diseases [7]. The association with SARS-CoV-2 has been reported in a 15-year-old patient who was diagnosed with anti-MOG IgG-associated bilateral optic neuritis a few days after COVID-19 illness [8].

Our patient is not the only one to have demonstrated a link between SARS-CoV-2 and visual impairment [9]. In that reported case, the association with demyelinating parenchymal lesions and elevated protein count in CSF were supportive evidence of inflammatory or dysimmune disease of the central nervous system. The possibility of a direct central nervous system infection (CNS) cannot be eliminated despite the negative SARS-CoV-2 PCR result test in the CSF [10]; however, it does suggest that this presentation was likely due to an immune-mediated inflammatory process rather than a direct invasion of SARS-CoV-2 into the CNS. The post-infectious autoimmune origin was very probable in our reported case after other causes were eliminated by an exhaustive assessment. The interesting feature, in that case, was the optic nerve’s normal MRI scans (1.5-Tesla magnet) on at least two occasions, which was a misleading factor. MRI-negative optic neuritis has been reported in 14% [11].

Post-COVID-19 dysimmune disease has been elucidated, but optic neuritis post-COVID was related to the presence of MOG antibodies in the case reported by Peters et al. [12].

The case of a patient with right optic neuritis following SARS-CoV-2 infection and demyelinating lesions in the CNS was also reported and was diagnosed then with multiple sclerosis [13]. The association between optic neuritis and unilateral panuveitis was reported by Benito-Pascual et al. as the first manifestation of a SARS-CoV-2 infection prior to the onset of pulmonary symptoms [14]. Whether SARS-CoV-2 is involved in initiating or exacerbating the inflammatory process leading to demyelinating diseases remains to be elucidated [15]. 

An additional case of optic neuritis that was associated with other neurological deficits and consistent with acute disseminated encephalomyelitis (ADEM) has also been documented [16].

Treatment of optic neuritis post-SARS-CoV-2 is still a matter of controversy in the literature and should be tailored to the individual patient. The efficacy of high doses of intravenous methylprednisolone, plasmapheresis, and immunoglobulin in viral immune-mediated neurological disorders led some authors to use them in this pathology [17]. Recent studies have confirmed its efficacy [12, 17]. It has been observed that functional recovery depends much on clinical presentation. Abrupt, severe onset and rapid progression have the poorest prognosis [12-13, 17].

## Conclusions

Our clinical case presented with typical symptoms of acute bilateral optic demyelinating neuritis. All paraclinical investigations, in particular, MRI and lumbar puncture, were unable to conclude with a diagnosis of multiple sclerosis or other autoimmune diseases. However, we believe in the existence of a causal link between the COVID-19 infection and the occurrence of blindness, via a dysimmunity mechanism. COVID-19 infection is still obscure and is not limited to simple respiratory disease, hence the interest of our publication which enriches the scientific community in terms of extrapulmonary COVID-19 disease.
